# Near Complete Repair After Myocardial Infarction in Adult Mice by Altering the Inflammatory Response With Intramyocardial Injection of α-Gal Nanoparticles

**DOI:** 10.3389/fcvm.2021.719160

**Published:** 2021-08-25

**Authors:** Uri Galili, Zhongkai Zhu, Jiwang Chen, Josef W. Goldufsky, Gary L. Schaer

**Affiliations:** ^1^Department of Medicine, Rush University Medical Center, Chicago, IL, United States; ^2^Department of Medicine, University of Illinois at Chicago, Chicago, IL, United States

**Keywords:** myocardial infarction, myocardial repair, mice, macrophages, anti-Gal antibody, α-gal epitope, α-gal nanoparticles

## Abstract

**Background:** Neonatal mice, but not older mice, can regenerate their hearts after myocardial-infarction (MI), a process mediated by pro-reparative macrophages. α-Gal nanoparticles applied to skin wounds in adult-mice bind the anti-Gal antibody, activate the complement cascade and generate complement chemotactic peptides that recruit pro-reparative macrophages which are further activated by these nanoparticles. The recruited macrophages decrease wound healing time by ~50%, restore the normal skin structure and prevent fibrosis and scar formation in mice.

**Objectives:** The objective of this study is to determine if α-gal nanoparticles injected into the reperfused myocardium after MI in adult-mice can induce myocardial repair that restores normal structure, similar to that observed in skin injuries.

**Methods and Results:** MI was induced by occluding the mid-portion of the left anterior descending (LAD) coronary artery for 30 min. Immediately following reperfusion, each mouse received two 10 μl injections of 100 μg α-gal nanoparticles in saline into the LAD territory (*n* = 20), or saline for controls (*n* = 10). Myocardial infarct size was measured by planimetry following Trichrome staining and macrophage recruitment by hematoxylin-eosin staining. Left ventricular (LV) function was measured by echocardiography. Control mice displayed peak macrophage infiltration at 4-days, whereas treated mice had a delayed peak macrophage infiltration at 7-days. At 28-days, control mice demonstrated large transmural infarcts with extensive scar formation and poor contractile function. In contrast, mice treated with α-gal nanoparticles demonstrated after 28-days a marked reduction in infarct size (~10-fold smaller), restoration of normal myocardium structure and contractile function.

**Conclusions:** Intramyocardial injection of α-gal nanoparticles post-MI in anti-Gal producing adult-mice results in near complete repair of the infarcted territory, with restoration of normal LV structure and contractile function. The mechanism responsible for this benefit likely involves alteration of the usual inflammatory response post-MI, as previously observed with regeneration of injured hearts in adult zebrafish, salamanders and neonatal mice.

## Introduction

Myocardial infarction (MI) is the cause of 25% of the deaths in the USA, primarily because of the extremely limited regenerative capacity of the myocardium (1). Myocardial damage post-MI usually heals by the default repair mechanism of fibrosis and scar formation which prevents subsequent rupture of the injured ventricular wall. This repair mechanism often results in reduced contractility which can lead to heart failure and premature death (2). Several studies of repair after MI in mice have suggested that this repair and healing mechanism is similar to that mediating healing and scar formation in skin wounds (3–8). In both healing events, “pro-inflammatory” polarized macrophages are the first to reach the injury and debride it of dead cells. Subsequently, “pro-reparative” polarized macrophages secrete cytokines which orchestrate angiogenesis, fibrosis and scar formation. In contrast to this repair mechanism, several vertebrates were found capable of natural regeneration of the injured myocardium, thereby restoring the original structure of the tissue without fibrosis. These include adult zebrafish (9), adult amphibians, such as salamander (10) and axolotl (11) and neonatal mice (12, 13) and pigs (14, 15). If injury to the heart in these mammalian neonates is caused during the first or second day after birth, the injured myocardium regenerates into its original structure, whereas injuries caused several days after birth result in fibrosis and scar formation as in the adult animal. These myocardial regeneration processes in fish, amphibians and mammalian neonates were found to be associated with extensive infiltration of macrophages into the injured tissue (16–20) and activation of the complement system (20–23). We have aimed to determine whether it is possible to induce by immunological means, extensive activation of the complement system and recruitment of macrophages into injured myocardium of post-MI adult mice, in order to induce myocardial repair similar to that observed in adult fish, amphibians and neonatal mice.

We have previously reported that extensive immune mediated complement activation which results in macrophage recruitment, is associated with accelerated regeneration and prevention of fibrosis in skin injuries of adult mice treated with α-gal nanoparticles (24–29). These nanoparticles present a carbohydrate antigen, called the “α-gal epitope,” with the structure Galα1-3Galβ1-4GlcNAc-R (25, 27). α-Gal epitopes bind the natural anti-Gal antibody which is abundant in all humans and constitutes as much as ~1% of immunoglobulins (30–33).

All mammals that are not monkeys or apes synthesize the α-gal epitope. Among primates, lemurs (evolved in Madagascar) and New World monkeys (monkeys of South America) also synthesize the α-gal epitope (30, 34, 35). All mammals synthesizing α-gal epitopes cannot produce the anti-Gal antibody since the α-gal epitope is a self-antigen in them. In contrast, humans, apes and Old-World monkeys (monkeys of Asia and Africa) lack α-gal epitopes and produce the natural anti-Gal antibody without active immunization, in response to constant antigenic stimulation by gastrointestinal bacteria (30–36). The reason for these differences in α-gal epitope synthesis in mammals is the differential activity of the α1,3galactosyltransferase (α*1,3GT*) gene (also called *GGTA1*) which codes the α1,3GT enzyme that synthesizes α-gal epitopes. This gene is active in all mammals synthesizing α-gal epitopes but has been evolutionary inactivated in ancestral apes and Old-World monkeys, thus it is inactivated in humans, as well (34, 35, 37).

Since anti-Gal is present in all humans and anti-Gal/α-gal immune complexes effectively activate the complement system, we hypothesized that formation of such immune complexes in the form of anti-Gal interaction with α-gal nanoparticles may be considered as a platform for future induction of a variety of regenerative therapies (30). The previous studies on skin injury repair and regeneration (24–29) indicated that anti-Gal/α-gal nanoparticles interaction at the administration site of these nanoparticles, activates the complement system to generate large amounts of C5a and C3a complement cleavage peptides that induce recruitment of multiple macrophages into the treated injuries. The recruited macrophages bind *via* their Fc receptors the Fc “tail” of anti-Gal immunocomplexed with the multiple α-gal epitopes on the nanoparticles and are activated to polarize into pro-reparative macrophages that secrete a variety of cytokines which decrease the healing time by ~50% and prevent fibrosis and scar formation.

**Figure 1:**
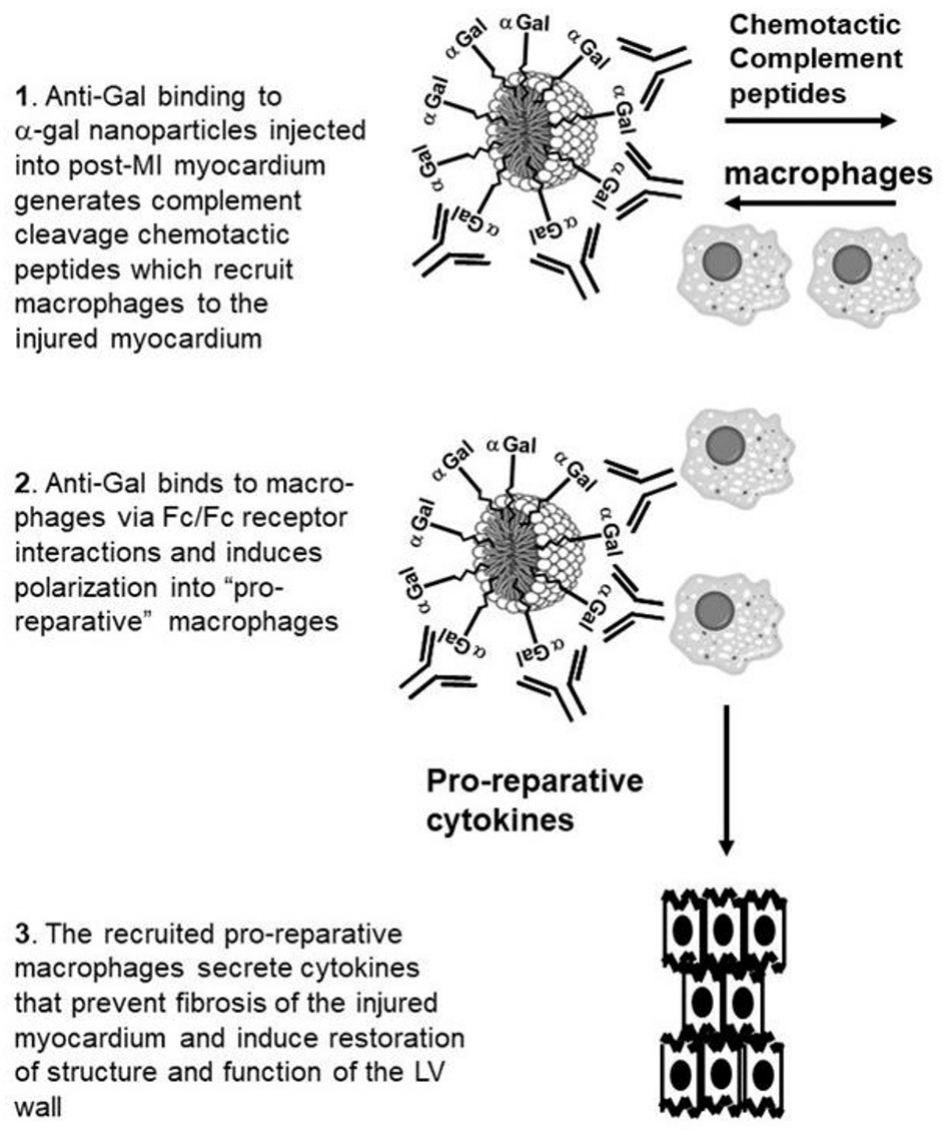
Hypothesis on post-MI myocardial repair by intramyocardial injection of α-gal nanoparticles: **Stage 1**. Anti-Gal binding to injected α-gal nanoparticles activates the complement system to generate chemotactic peptides that recruit macrophages. **Stage 2**. Recruited macrophages bind *via* Fc-receptors the Fc “tail” of anti-Gal coating the α-gal nanoparticles and are induced to polarize into macrophages secreting pro-reparative cytokines. **Stage 3**. The pro-reparative cytokines induce structure and function restoration of the injured myocardium.

In the present study we hypothesized that α-gal nanoparticles may contribute to repair of post-MI mouse heart and prevent scar formation, as illustrated in Figure 1. The various stages of the repair process, as hypothesized in adult mouse heart, are as follows: **Stage 1**. Post-MI injection of α-gal nanoparticles into the injured myocardium of mice producing anti-Gal will result in anti-Gal/α-gal nanoparticles interaction which activates the complement system to generate the complement cleavage chemotactic peptides C5a and C3a that recruit macrophages. **Stage 2**. Recruited macrophages bind *via* their Fc-receptors the Fc “tail” of anti-Gal coating the α-gal nanoparticles and are induced to polarize into macrophages secreting pro-reparative cytokines. **Stage 3**. The pro-reparative cytokines induce restoration of structure and function of the injured myocardium.

The study of this hypothesis required a unique strain of knockout mice (called GT-KO mice) in which the α1,3galactosyltransferase gene (α*1,3GT* gene also called *GGTA1*) is disrupted (38). GT-KO mice do not produce anti-Gal unless they are immunized with immunogenic glycoproteins (e.g., xenoglycoproteins) presenting multiple α-gal epitopes. The immunogenic glycoproteins activate helper T cells which cannot be activated by α-gal epitopes (39). Pig kidney membrane (PKM) homogenate of wild-type pigs were used as an immunogen for this purpose since glycoproteins in these cell membranes present large amounts of α-gal epitopes (39–41). The mice require such immunization for producing anti-Gal because they live in a sterile environment that does not enable the development of a gastrointestinal flora, which in humans induces production of the natural anti-Gal antibody (25, 30, 31, 39). MI was performed in the mice by an occlusion/reperfusion procedure. Here we demonstrated near complete repair of the ischemia injured myocardium following intramyocardial injection of α-gal nanoparticles in post-MI adult mouse hearts.

## Methods

### Preparation of α-Gal Nanoparticles

α-Gal nanoparticles were prepared as previously described (24, 25, 27) from rabbit red blood cell (RBC) membranes because these RBC present the highest concentration of α-gal epitopes in comparison to other mammalian RBC and a large proportion of these α-gal epitopes is presented on glycolipids (34). Rabbit RBC (1 L) are lysed in water and washed for hemoglobin removal. Washed RBC membranes are mixed with 800 ml chloroform and 800 ml methanol for 2 h, then 800 ml methanol are added and stirred overnight, resulting in the extraction of the phospholipids, cholesterol and glycolipids into the chloroform:methanol solution (24, 25, 40). Residual RBC membranes and proteins are precipitated by the chloroform:methanol solution. The precipitate is removed by filtration through Whatman paper. The extract is dried in a rotary evaporator, weighed, and sonicated in saline, in a sonication bath to generate a suspension of 100 mg/ml liposomes comprised of phospholipids, cholesterol and glycolipids. Residual debris is pelleted at low speed centrifugation (800 rpm) and removed. Liposomes are further sonicated on ice with a sonication probe to break the liposomes into submicroscopic liposomes (50–300 nm), referred to as “α-gal nanoparticles,” which are sterilized by filtration through a 0.45 μm filter (Millipore). These nanoparticles present ~10^15^ α-gal epitopes per mg nanoparticles (25). Nanoparticles lacking α-gal epitopes were produced by the same method from RBC of knockout pigs for the α1,3galactosyltransferas gene (GT-KO pigs) that cannot synthesize α-gal epitopes (42, 43).

### Mouse Experimental Model

The experimental protocol was approved by the Institutional Animal Care and Use Committee at Rush University Medical Center (Chicago, IL) and performed in accordance with AAALAC guidelines. In order to simulate a human-like immune environment, a previously established, α1,3galactosyltransferase (α1,3GT) knockout mouse (GT-KO) was used (38). Like humans, these knockout mice do not synthesize the α-gal epitope and therefore can produce the anti-Gal antibody with post-natal exposure to this epitope, such as immunization with PKM homogenate (24, 25, 39, 40). PKM were empirically found to be more effective than rabbit RBC in immunization of GT-KO mice for the production of the anti-Gal antibody (unpublished observations). GT-KO mice used (males and females, age 12–16 weeks) were of C57BL/6 × BALB/c genetic background (38). Mice were induced to produce the anti-Gal antibody at titers comparable to those in humans by 5 weekly immunizations with 50 mg PKM homogenate (200 mg/ml). These homogenates were used for immunization that elicits anti-Gal production because pig kidney membranes present a large number of α-gal epitopes (41). In the absence of such immunization, GT-KO mice do not produce anti-Gal because of lack of immunizing gastrointestinal bacteria (39).

### Induction of MI and Injection of α-Gal Nanoparticles

MI induction with subsequent coronary reperfusion was performed in mice based on modification of previously published protocols (44, 45). Mice were anesthetized with ketamine (100 mg/kg)/xylazine (5 mg/kg) injected intraperitoneal, intubated and anesthesia maintained by inhaled 1.0% isoflurane. A left thoracotomy was performed *via* the fourth intercostal space and lungs were retracted to expose the heart. The mid-LAD coronary artery was ligated with a 7-0 silk suture. Ligation was confirmed as 100% occlusive by the appearance of pallor of the anterior wall of the left ventricle (LV). After 30 min occlusion, the ligature was removed, permitting reperfusion of the LAD territory. This was confirmed by noting a change in the color of the anterior wall of the LV from pallor to deep red, as observed prior to coronary occlusion. One minute after reperfusion, a 32-gauge needle was used to inject 10 μl of a 10 mg/ml α-gal nanoparticles suspension in saline [dose optimized in refs. (24, 25)] into the LV anterior wall, ~1 mm distal to the LAD ligation site. Treated mice received two injections, 1–2 mm apart (100 μg nanoparticles per site). Control mice underwent the same procedure but received two injections of 10 μl normal saline. Subsequently, the chest wall, subcutaneous tissue, and skin were sutured. Among treated animals, 20 underwent post-mortem studies on day 28 post-MI, and 2 animals per day at 4, 7, and 14 days post-MI. Among controls, 10 mice underwent post-mortem studies on day 28 post-MI, and 2 animals per day, 4, 7, and 14 days post-MI. Mice were euthanized by CO_2_ inhalation followed by cervical translocation.

### Histologic Specimen Preparation

Formalin fixed hearts were cut from mid-level (at the level of the LAD occlusion) into 5 equal double sections (5 μm-thick) 300 μm apart. Sections at each level were stained with either hematoxylin-eosin (H&E) (to assess macrophage infiltration), or Trichrome (collagen/fibrosis stain blue; viable cardiomyocytes stain red; debrided cardiomyocyte areas stain gray, RBCs stain brown). The LV section demonstrating the greatest extent of macrophage infiltration or the greatest infarct size (out of the sections prepared at 5 levels), was scanned for planimetry measurement (46) using AperioImageScope planimetry program. The scanned histology slides were measured for infarct size in a blinded manner according to a code number which subsequently was compared with a key list which indicated the treatment of each heart. The infarct size for each scanned section was expressed as percentage, relative to the total area of uninjured LV (46). Sections were also stained with antibody to F4/80 antigen for identification of macrophages (25) and with antibodies to proliferating cell nuclear antigen (PCNA) (Abcam, Cambridge, MA) for detection of proliferating cardiomyocytes. The staining was performed following antigen retrieval with Tris-EDTA buffer (10 mM Tris Base, 1 mM EDTA solution, 0.05% Tween 20, pH 9.0, kept for 10 min in boiling solution) and completed with a fluorescein coupled secondary antibody. After washes the slides were counterstained with DAPI.

### Assessment of LV Function With Transthoracic Echocardiography

High resolution transthoracic echocardiography (Vevo 770, VisualSonics) was performed in mice (*n* = 4 treated, *n* = 4 controls) that were sedated with 1% isoflurane, before LAD ligation, and post-MI (at 7 and 28 days). Parasternal short-axis views at the mid-ventricular level were obtained for M-mode analysis of the LV internal diameter at end diastole (LVDD) and end systole (LVDS). Fractional shortening (FS) was calculated as: %FS = [(LVDD – LVDS)/LVDD] × 100.

### Staining for Proliferating Cells by Nuclear BrdU Uptake

Mice were injected intraperitoneally with 1.0 ml of a solution containing 1 mg BrdU (Sigma-Aldrich, St. Louis, MO). The injection was performed on Day 9 post-MI or on Day 11 post-MI. The mice were euthanized after 24 and 48 h, heart and intestine were harvested fixed in formalin and embedded in paraffin. The intestine was used as positive control for each of the hearts studied. Paraffin sections were deparaffinized and subjected to BrdU nuclear staining by the use of “BrdU *in situ* Detection Kit” (BD Pharmingen cat. No. 550803), according to the manufacturer instruction. The kit includes biotinylated anti-BrdU antibody and avidin coupled peroxidase. The color reaction is achieved by diaminobenzidine (DAB) precipitation. Counter staining was performed with hematoxylin.

### Statistics

The data were presented as mean ± SE. Comparisons between 2 groups were made using Student *t*-test. Multiple group comparisons were made using One-way ANOVA, followed by Tukey's multiple comparison test.

## Results

### Effects of Anti-Gal Interaction With α-Gal Nanoparticles

**Figure 2:**
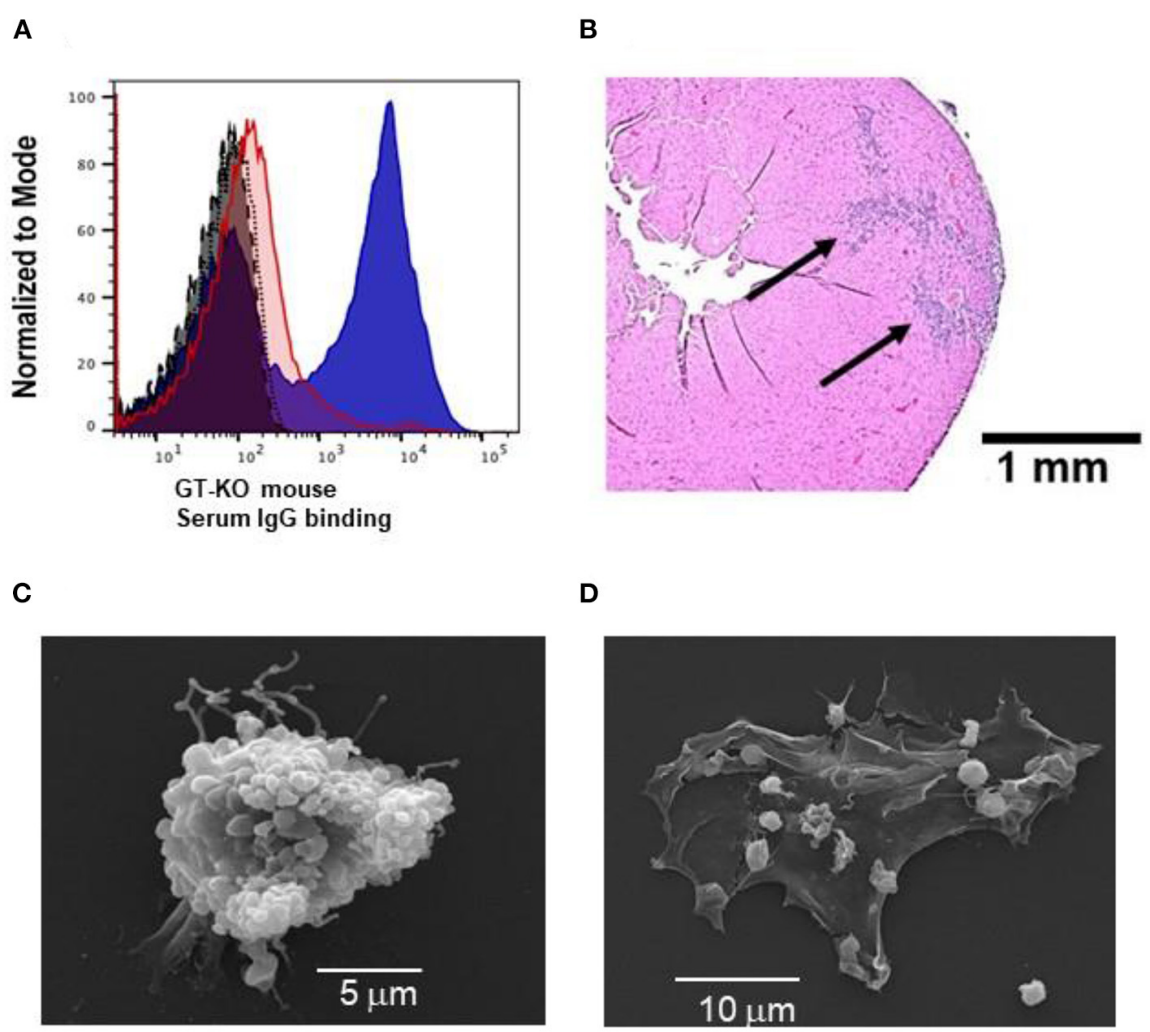
Characteristics of anti-Gal interaction with α-gal nanoparticles: **(A)** Flow cytometry evaluation of anti-Gal IgG binding to α-gal nanoparticles (blue) (representing Stage 1 of Figure 1); nanoparticles lacking α-gal epitopes (red), isotype-control (gray), auto-fluorescence without antibodies (black). **(B)** *In vivo* recruitment of macrophages by α-gal nanoparticles, 4 days post-injection of the nanoparticles into normal uninjured mouse heart (also representing Stage 1 of Figure 1). The section is in a representative heart of 4 hearts with similar results. Arrows – macrophages infiltrating two injection sites. Staining by hematoxylin-eosin (H&E). **(C)** Scanning electron microscopy of anti-Gal-coated α-gal nanoparticles binding to a macrophage (representing Stage 2 in Figure 1). **(D)** As **(C)** however the initial concentration of the applied α-gal nanoparticles is 100-fold lower, thus the cell membrane of the macrophage is visible.

Some of the outcomes of anti-Gal interaction with α-gal nanoparticles, hypothesized in Stages 1 and 2 in Figure 1 are illustrated in Figure 2. Anti-Gal within the serum of GT-KO mice producing this antibody readily binds to α-gal epitopes on α-gal nanoparticles, as shown by flow cytometry in Figure 2A. Nanoparticles lacking α-gal epitopes (produced from RBC of GT-KO pigs that lack these epitopes) do not bind anti-Gal produced by GT-KO mice. The binding of anti-Gal to α-gal nanoparticles was previously shown to result in activation of the complement system and production of complement cleavage chemotactic peptides that effectively induce macrophage recruitment to the nanoparticles injection sites in the uninjured skin of GT-KO mice (25). A similar recruitment of macrophages could be demonstrated by two injections (each of 10 μl of the α-gal nanoparticles) into uninjured LV myocardium of healthy hearts, not subjected to LAD occlusion. This is shown in a representative heart evaluated 4 days post-injection (Figure 2B). High power magnification inspection of the section in Figure 2B and of the other 3 uninjured normal hearts that received the same injection technique revealed macrophages and no polymorphonuclear cells or fibroblasts among the infiltrating cells. Similar infiltration of only macrophages (indicated as F4/80 stained cells) was previously observed in mouse dermis injected with α-gal nanoparticles (25, 27). Injection of saline into uninjured hearts resulted in no infiltration of macrophages or other cells at any time point (not shown). The similarity in recruitment of macrophages in uninjured skins (25) and hearts injected with α-gal nanoparticles (Figure 2B), strongly suggests that complement cleavage chemotactic peptides produced as a result of anti-Gal/α-gal nanoparticles interaction, shown to mediate recruitment of macrophages in the skin (25) have a similar chemotactic effect in post-MI reperfused hearts injected with these nanoparticles, as illustrated in Stage 1 of Figure 1.

The predicted Fc/Fc receptor interaction between anti-Gal coated α-gal nanoparticles and recruited macrophages (Stage 2 in Figure 1) is shown in Figure 2C with macrophages incubated for 2 h at 24°C with 10 mg/ml of such nanoparticles. The representative macrophage is covered with the bound α-gal nanoparticles that are coated with anti-Gal. The binding of anti-Gal coated α-gal nanoparticles also causes the contraction of the macrophage into a more spherical shape because of the multiple Fc/Fc receptor interactions. When the nanoparticles concentration is 100-fold less (i.e., 0.1 mg/ml) fewer nanoparticles bind to the macrophage, thus the macrophage maintains a normal flattened morphology (Figure 2D). In the absence of anti-Gal, no nanoparticles were bound to the macrophages (not shown).

### Histological Evidence of Myocardial Repair With α-Gal Nanoparticles

**Figure 3:**
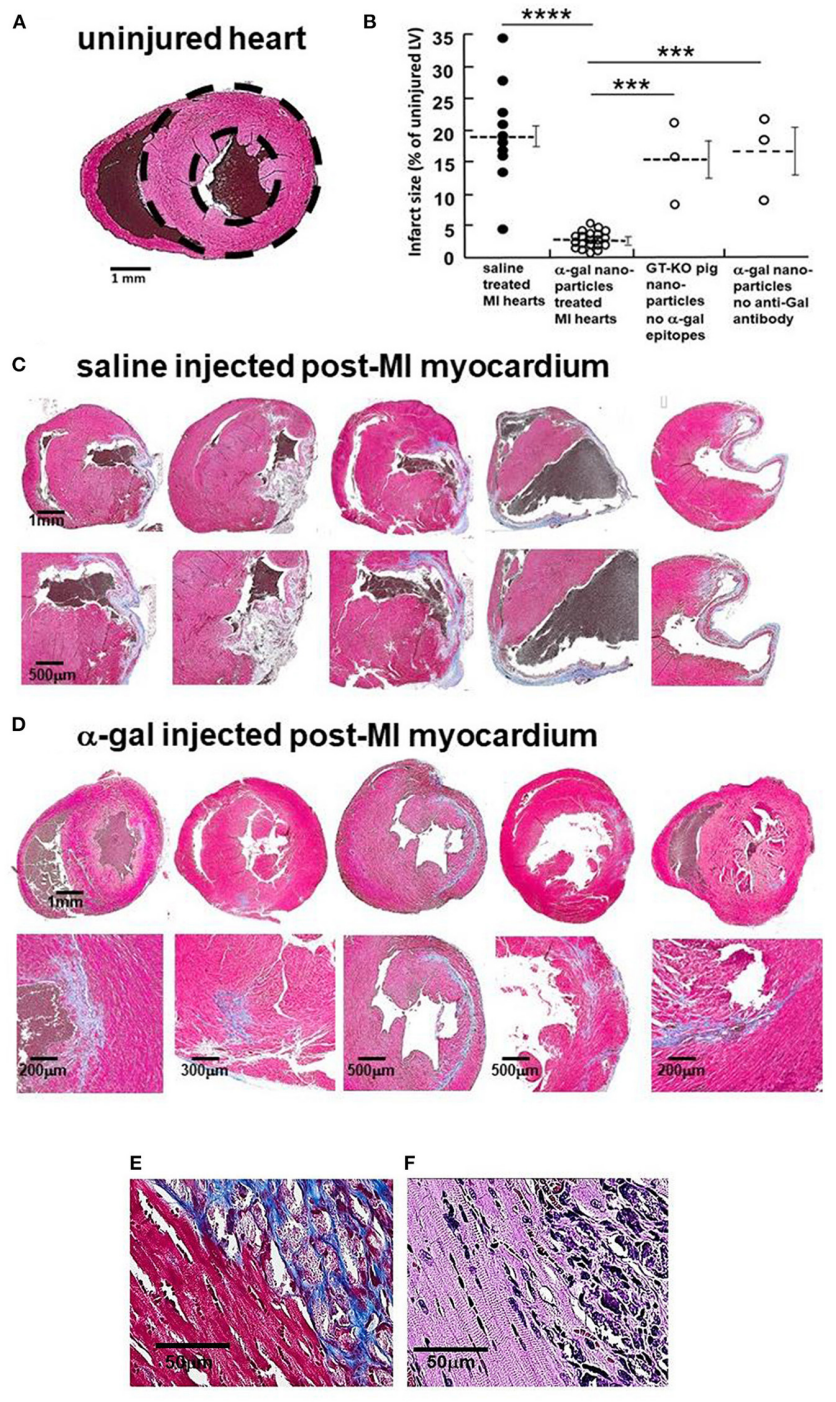
Myocardial repair 28 days post-MI in adult mice treated with α-gal nanoparticles. **(A)** Normal heart- dashed circles demarcate LV (Trichrome, uninjured cardiomyocytes-red, RBCs-brown). **(B)** Infarcted size determined by fibrosis due to myocardial infarction, measured by planimetry and calculated as percentage, relative to the area of uninjured LV in 10 saline controls, 20 α-gal nanoparticles treated hearts, 3 hearts injected with nanoparticles lacking α-gal epitopes, and 3 hearts from mice lacking anti-Gal and treated with α-gal nanoparticles. Results are also presented as mean (dashed lines) ±SE. Horizontal solid lines indicate statistical comparisons between groups. Statistical analysis was performed by One-way ANOVA, followed by Tukey's multiple comparison test, ****p < 0.0001; ***p < 0.001. **(C–E)** Trichrome staining (collagen/fibrosis stains-blue, uninjured cardiomyocytes-red, RBCs-brown). **(C)** Saline treated hearts (additional 5 in Supplementary Figure 1A). Fibrosis areas magnified in lower figure of each pair. **(D)** α-Gal nanoparticles treated hearts (additional 15 in Supplementary Figure 1B). Fibrosis areas magnified in lower figure of each pair. **(E)** Border between fibrotic tissue and healthy appearing cardiomyocytes in α-gal nanoparticles treated hearts. **(F)** H&E staining of E, demonstrating sarcomeric striation within cardiomyocytes of normal appearance [in **(E,F)**, one representative of 10 hearts with similar results].

The hypothesized Stage 3 in Figure 1 suggests that following the Fc/Fc receptor interactions between the recruited macrophages and anti-Gal coated α-gal nanoparticles in injured myocardium, the macrophages polarize into pro-reparative macrophages that secrete cytokines which, similar to their effects in wound healing, enhance repair and restore the normal structure and function of the post-MI myocardium. These hypothesized effects were studied in GT-KO mouse hearts assessed 28 days after the 30 min of mid-LAD occlusion and reperfusion in mice treated with α-gal nanoparticles, and saline-treated controls. The normal mouse heart (not subjected to LAD occlusion) (Figure 3A) demonstrates the expected uniform Trichrome red staining of uninjured cardiomyocytes and no fibrosis. Dashed circles mark epicardial and endocardial surfaces of the LV. The area between the circles represents 100% uninjured LV. The right ventricle (when visible) is on the left, and the LV on the right of each figure. The size of myocardial infarct (Trichrome staining collagen/fibrosis blue) was calculated as percentage, relative to the area of uninjured LV, based on planimetry measurements (46) of the injured and non-injured LV (Figure 3B). The histological sections of hearts providing the data for Figure 3B are presented in Figures 3C,D and in Supplementary Figure 1.

In saline injected controls (Figure 3C; Supplementary Figure 1A), mid-LAD occlusion for 30 min followed by reperfusion for 28 days resulted in large transmural infarcts with extensive fibrosis, scar formation and wall thinning. The infarct size, extent of fibrosis and patterns of scar formation are highly variable among the 10 control animals, in which the % mean ± SE infarct size of the LV was 19 ± 2.3%, with a range of 5–35% - (Figure 3B). This variability may result from small differences in the site of LAD occlusion and amount of myocardium supplied distal to the occlusion. Similar distribution of infarct size following 30 min occlusion/reperfusion in mice also was observed by other investigators (47–49). Twenty mice treated with α-gal nanoparticles demonstrated dramatically smaller infarcts, with greatly less fibrosis and thinning of the LV wall (Figure 3D; Supplementary Figure 1B). The mean myocardial infarct size in treated animals was 2.2 ± 1.2%, with a range of 0.1–5.0% (Figure 3B). The tissue stained red surrounding the residual blue fibrotic tissue in α-gal nanoparticles treated hearts (Figure 3E) is comprised of normal appearing cardiomyocytes that display (under high magnification of H&E stain) characteristic sarcomeric striation (Figure 3F), with minimal fibrosis.

Two additional types of controls demonstrate that the reparative effects of α-gal nanoparticles are dependent on the specific interaction between the anti-Gal antibody and α-gal epitopes on the nanoparticles, as hypothesized in Figure 1. Injection of GT-KO pig nanoparticles (i.e., nanoparticles lacking α-gal epitopes) into the reperfused post-MI myocardium resulted in fibrosis and scar formation that is similar to mice treated with saline (Figure 3B). The individual hearts receiving this treatment are shown in Supplementary Figure 2A. Furthermore, injection of α-gal nanoparticles into the post-MI heart of GT-KO mice lacking anti-Gal (i.e., not immunized with pig kidney membranes homogenate) resulted in fibrosis and scar formation, as well (Figure 3B; Supplementary Figure 2B), rather than in extensive myocardial restoration of structure, observed in anti-Gal producing mice. Thus, both anti-Gal production and α-gal epitopes on the nanoparticles are required for the induction of the reparative response in injured myocardium of adult mice.

### Evidence of Restoration of LV Function With α-Gal Nanoparticles

**Figure 4:**
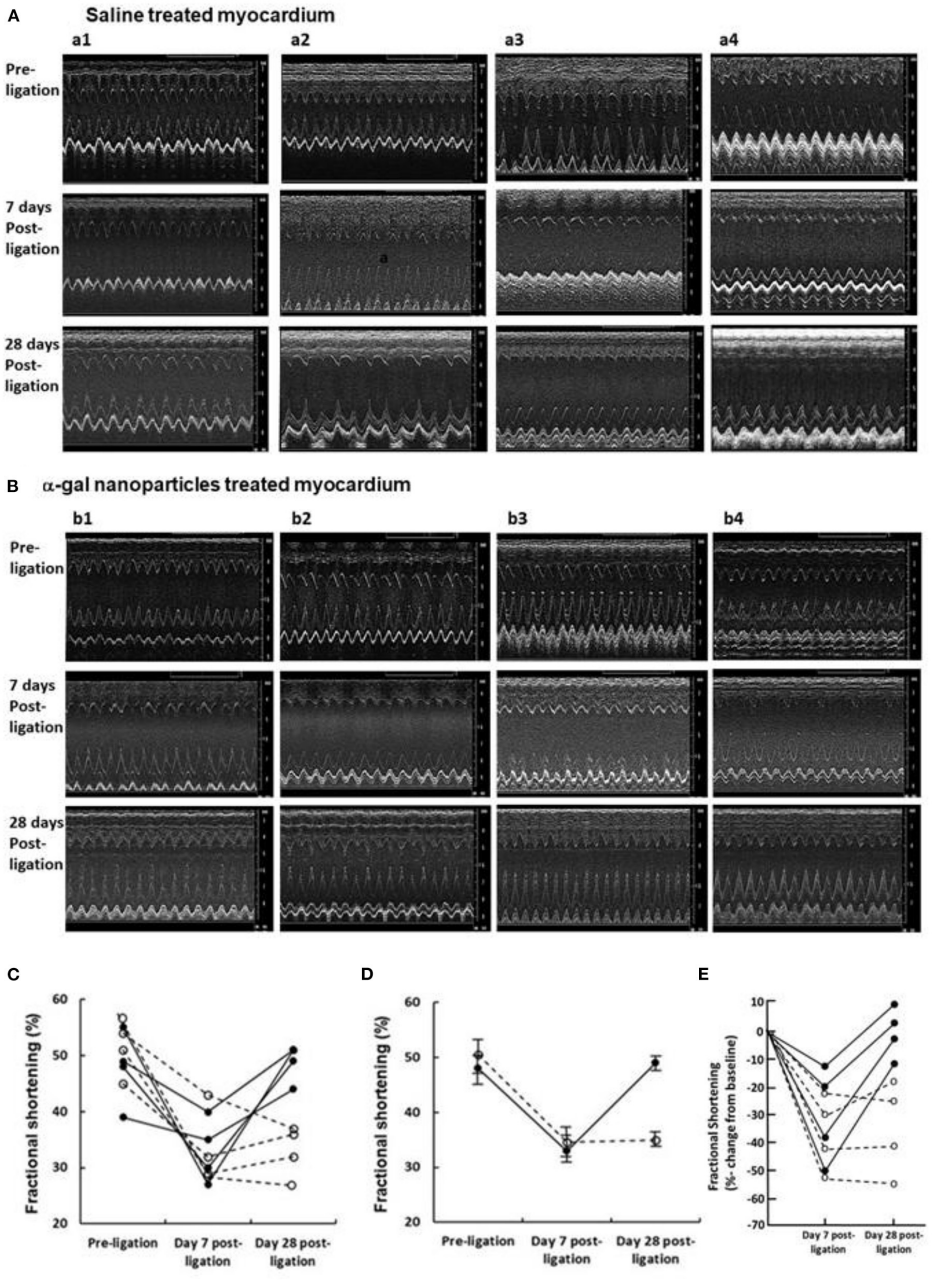
Echocardiography of **(A)** 4 saline controls (a1–4), and **(B)** 4 α-gal nanoparticles treated mice (b1–4), pre-LAD ligation, 7- and 28-days post-ligation. **(C)** Fractional-shortening (FS) at each 3 time points. Dashed-lines and open-circles represent control mice (a1–4), solid-lines and closed-circles represent treated mice (b1–4). **(D)** Mean ±SE at each time points. FS data for the two groups are significantly different only on Day 28. Statistical analysis was performed by Student *t*-test, *p* < 0.001. **(E)** Data presented as percentage changes from baseline in Fractional Shortening at 7- and 28-days post-ligation. Symbols and lines are as in **(C)**.

**Figure 5:**
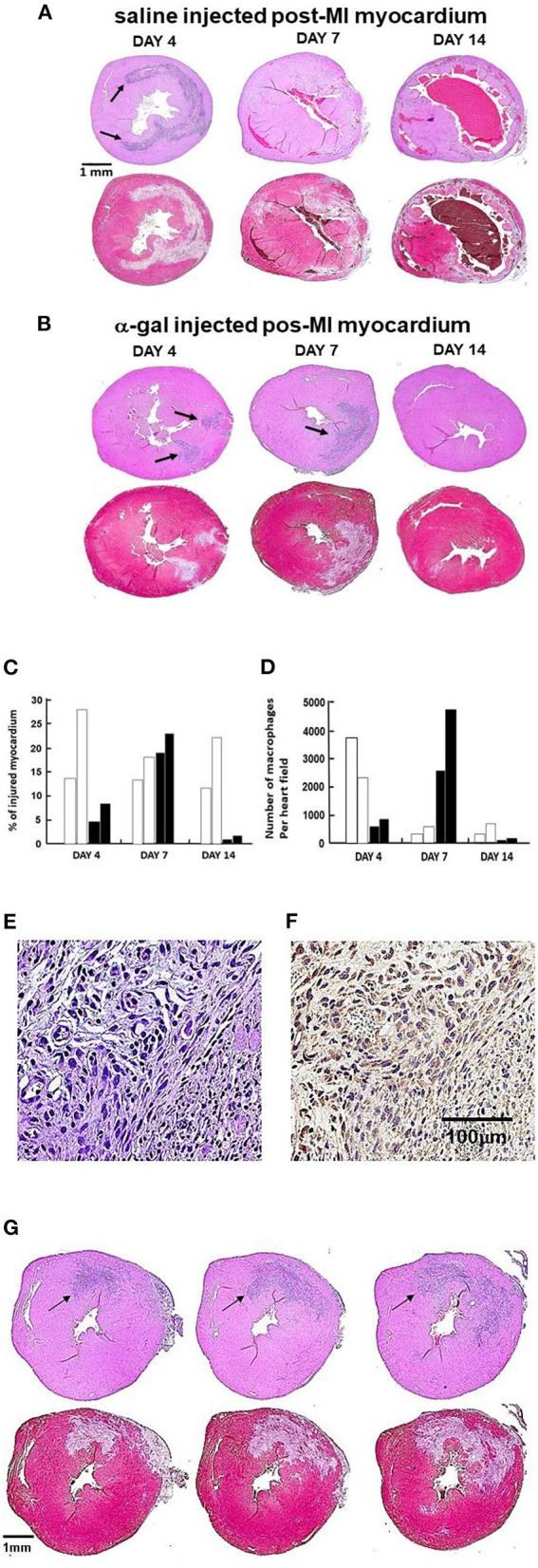
Post-MI injury and macrophage infiltration at 4, 7, 14 days post-MI in controls and treated mice (*n* = 2 at each time point). In **(A,B)**, the upper image of each pair is stained with H&E (arrows indicate areas of macrophage infiltration); lower image is stained with Trichrome (areas debrided of cardiomyocytes are stained gray). **(A)** One representative saline control at each of 3 time points post-MI. **(B)** One representative α-gal nanoparticles treated animal at each time point. The arrows mark macrophages infiltrating areas. Note on Day 4, arrows mark the macrophages at the two nanoparticles injection sites. **(C)** Planimetry of injured myocardium as percent of LV relative to the area of uninjured LV in two hearts per time-point. Open-columns: saline control: closed-columns: α-gal nanoparticles treatment. **(D)** Quantification of infiltrating macrophages within injured myocardium (H&E), columns as in **(C)**. **(E)** Staining of the infiltrating cells into heart injected with α-gal nanoparticles and viewed on Day 7 post-MI (H&E). **(F)** Cells in **(E)** are macrophages as they stain with peroxidase coupled anti-F4/80 antibody, an antibody that binds specifically to macrophages (Representative of three hearts with similar results). **(G)** Demonstration of the full overlap between the area with infiltrating macrophages (H&E staining) and the corresponding area debrided of injured cardiomyocytes (Trichrome staining). The sections to the left and right of the middle section (also shown at Day 7, **B**) are 300 μm above and below the middle section, respectively. The full overlap between areas of macrophages and debrided areas strongly suggests that the macrophages debride the damaged cardiomyocytes in the areas they reside.

Echocardiography was used to assess LV function in four saline treated controls (Figure 4A) and four α-gal nanoparticles treated mice (Figure 4B). Mice in both groups had normal fractional shortening (FS) before LAD occlusion (Figures 4C,D). By Day 7 post-MI, both groups demonstrated a marked reduction in FS, associated with LV chamber dilation, emphasizing the severity of the ischemic injury (Figures 4C–E). These Day 7 echocardiography results indicate that the post-ischemia injuries in both saline and α-gal nanoparticles treated hearts were severe and correlate with the histopathology results demonstrated below in Figure 5.

However, by Day 28, α-gal nanoparticles treated mice demonstrated a dramatic recovery of FS and return of LV chamber dimension to normal size, whereas control mice continued to display impaired FS and chamber dilation (Figures 4C–E). It is of note that the mice included in Figure 4 also provided histopathology results at Day 28. These results are included among the 10 saline treated mice which demonstrated fibrosis and scar formation and among 20 α-gal nanoparticles treated mice demonstrating repair and restoration of structure (Figure 3; Supplementary Figure 1) and are consistent with the functional results in Figure 4.

### α-Gal Nanoparticles Alter the Inflammatory Response Post-MI

The near-complete restoration of structure and function in the post-MI myocardium treated with α-gal nanoparticles, compared to the extensive fibrosis and scar formation in saline injected control hearts, raised the question whether there are observable differences in the time-course of the inflammatory response in the two groups. This was assessed by histopathology at 4, 7, and 14 days post-MI (Figure 5). Myocardial histopathology in one of two mice studied at each time point is shown in pairs of which the upper is stained with H&E and lower with Trichrome. In these sections, H&E staining identifies the infiltrating macrophages which are much smaller than the cardiomyocytes and lack the elongated shape and sarcomere striation, whereas Trichrome staining identifies areas debrided of injured cardiomyocytes (stained gray) and areas containing healthy cardiomyocytes (stained red). Planimetry measurements of the debrided areas in two mice at each time point are presented in Figure 5C and the number of observed macrophages in Figure 5D. The identity of the infiltrating cells as macrophages is demonstrated in Figures 5E,F by the staining with the macrophage specific F4/80 antibody.

At Day 4, the myocardium of control animals displayed extensive cardiomyocytes elimination (gray staining with Trichrome), coinciding with extensive infiltration of macrophages, similar to previous reports (4–8, 50–52). Hearts treated with α-gal nanoparticles displayed distinctly less macrophage infiltration at Day 4 (Figures 5B–D), suggesting an attenuated early inflammatory response. In accord with previous observations (52), the number of macrophages in control hearts greatly decreased within the injured myocardium at Day 7, whereas at Day 14, there was thinning and fibrosis of the LV wall and very low macrophage content (Figures 5A,C,D). In contrast to control mice, α-gal nanoparticles treated hearts demonstrated at Day 7 a marked increase in macrophage infiltration in the area debrided of cardiomyocytes. The areas in which cardiomyocytes died and were debrided in these mice are stained gray in Trichrome staining and comprise 19–24% of the LV myocardium (Figures 5B,C). The infiltrating cells on Day 7 were confirmed to be macrophages by positive *in situ* staining of the infiltrating mononuclear cells (Figure 5E, H&E) with the macrophage specific peroxidase-linked F4/80 antibody (Figure 5F). The areas in which macrophages infiltrated at Day 7 in the α-gal nanoparticles treated hearts, fully overlapped the areas devoid of cardiomyocytes (Figure 5B). This is further demonstrated in additional sequential sections at different planes of Day 7 α-gal nanoparticles treated heart (Figure 5G, middle section also shown in Figure 5B). Histological sections above and below the middle section (left and right figures, respectively) display a different distribution of infiltrating macrophages, suggesting that the injured area is of a substantial size with three-dimensional irregular shape. The overlap in the shape of the macrophage infiltrate (H&E) and the area devoid of cardiomyocytes (gray color in Trichrome staining) in Figure 5G, further suggests that a high proportion of the cardiomyocytes died following the ischemia and that one of the activities of macrophages recruited by α-gal nanoparticles is debriding areas of necrotic cardiomyocytes that were injured by the 30 min of LAD occlusion. Similar to control mice, very few macrophages were detected at Day 14 in α-gal nanoparticles treated hearts (Figures 5B,D). However, in clear contrast to control hearts which displayed at Day 14 thinning of the LV wall and fibrosis, α-gal nanoparticles treated hearts at Day 14 displayed near-complete restoration of myocardium structure and only minor residual fibrosis (Figures 5B,C). These observations strongly suggest that the areas debrided of cardiomyocytes at Day 7 in α-gal nanoparticles treated hearts were repopulated with healthy cardiomyocytes, as shown in hearts studied both at Days 14 and 28. It is of note that although the kinetics of macrophage infiltration differs between saline treated hearts and α-gal nanoparticles treated hearts, the damage caused to the myocardium (i.e., % infarct size) is not significantly different in both groups on Day 7. This observation is supported by the echocardiography studies displaying similar impaired contractility in the two groups on Day 7 (Figure 4). However, by Day 28 the contractility is restored to normal in the α-gal nanoparticles treated mice vs. continuation in impaired contractility in saline treated mice.

### Evaluating Cell Proliferation in α-Gal Nanoparticles Treated Hearts

**Figure 6:**
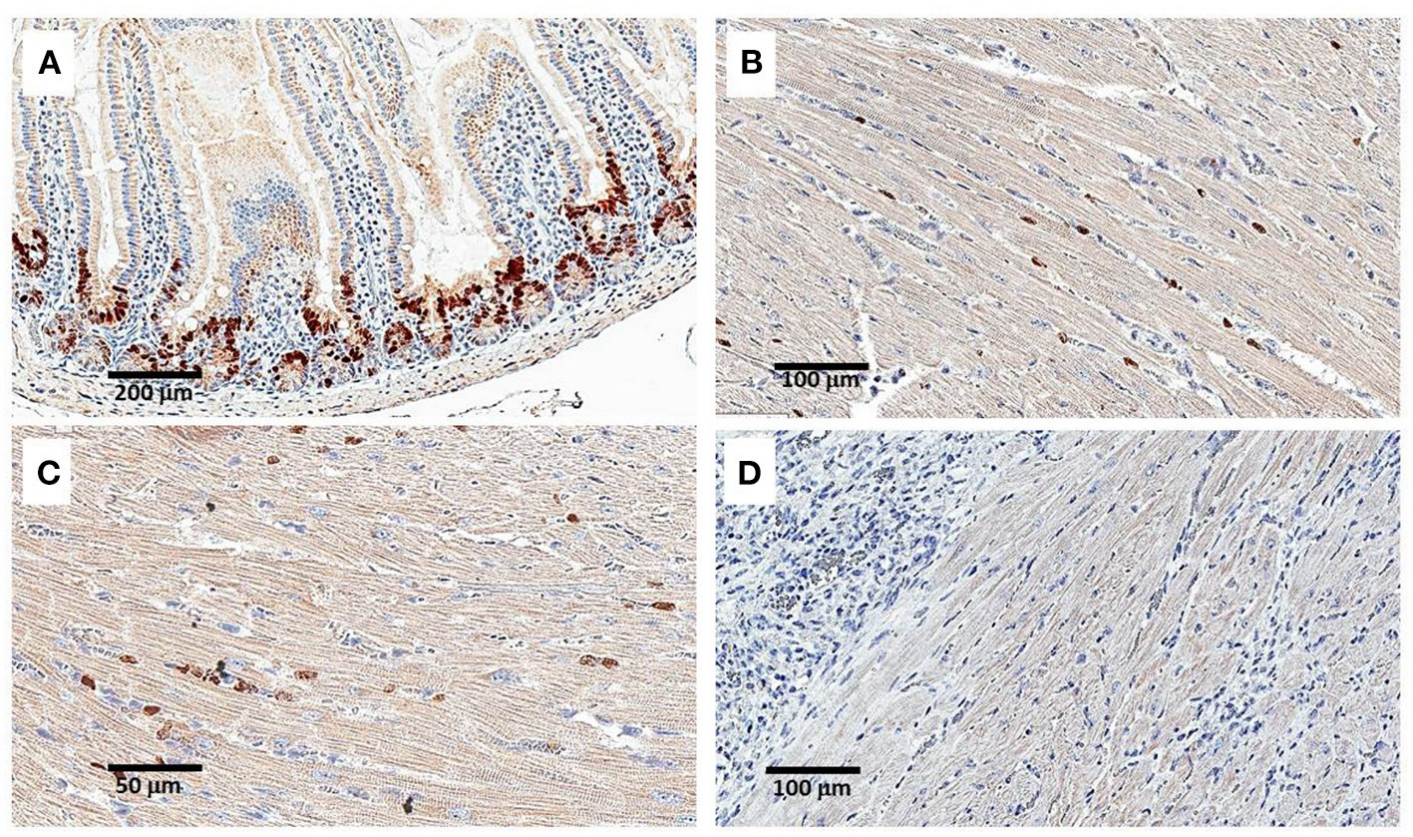
Staining for BrdU uptake by proliferating cells, 24 h following intraperitoneal injection of 1 mg BrdU. Injection was performed on Day 9 post-MI and intramyocardial α-gal nanoparticles injection. **(A)** Intestinal villi demonstrating effective uptake of BrdU in proliferating cells at the base of the villi (serving as a positive control). **(B–D)** Staining of the LV for BrdU uptake in an α-gal nanoparticles treated mouse. Only sparse uptake is observed. In **(D)** the section displays an area of macrophage infiltration near the cardiomyocytes. Data from one mouse of 5 with similar results.

The histological studies on post-MI myocardial repair presented in Figure 5 imply that the repopulation by cardiomyocytes following injection of α-gal nanoparticles occurs in the second week post-treatment. This raised the question whether the repopulation of the injured myocardium with healthy cardiomyocytes is the result of proliferation of pre-existing cardiomyocytes, or of resident stem/progenitor cells. This was studied on Days 9 and 11 post-MI in mice treated with α-gal nanoparticles by evaluating BrdU (thymidine analog) uptake into nuclei of proliferating cells in the heart 24 and 48 h after intraperitoneal injection of 1 mg BrdU. The sections were stained overnight with the biotinylated anti-BrdU antibody followed by avidin-peroxidase and DAB precipitation. Figure 6 shows the results in a representative mouse studied 24 h post-injection of BrdU on Day 9 (out of 5 mice with similar results). BrdU was effectively taken up by nuclei of proliferating cells at the base (crypt) of intestinal villi of the mice, which served as positive controls of proliferating epithelium (Figure 6A). However, in the heart, the staining of nuclei was sparse and mostly of the stained nuclei that did not correspond to distinct cardiomyocytes (Figures 6B,C). In view of distinct areas of injured cardiomyocytes debrided by macrophages (Figure 5), it would be expected that repopulation of these debrided areas by cells proliferating within the heart will result in appearance of groups of cardiomyocytes displaying uptake of BrdU. Similarly, no such BrdU staining pattern has been observed in any of the hearts in mice injected with BrdU on Day 9 and evaluated 48 h later, or in mice injected on Day 11 and evaluated after 24 or 48 h (not shown). In addition, no distinct nuclear BrdU staining was detected in cardiomyocytes in regions adjacent to areas containing infiltrating macrophages (Figure 6D).

**Figure 7:**
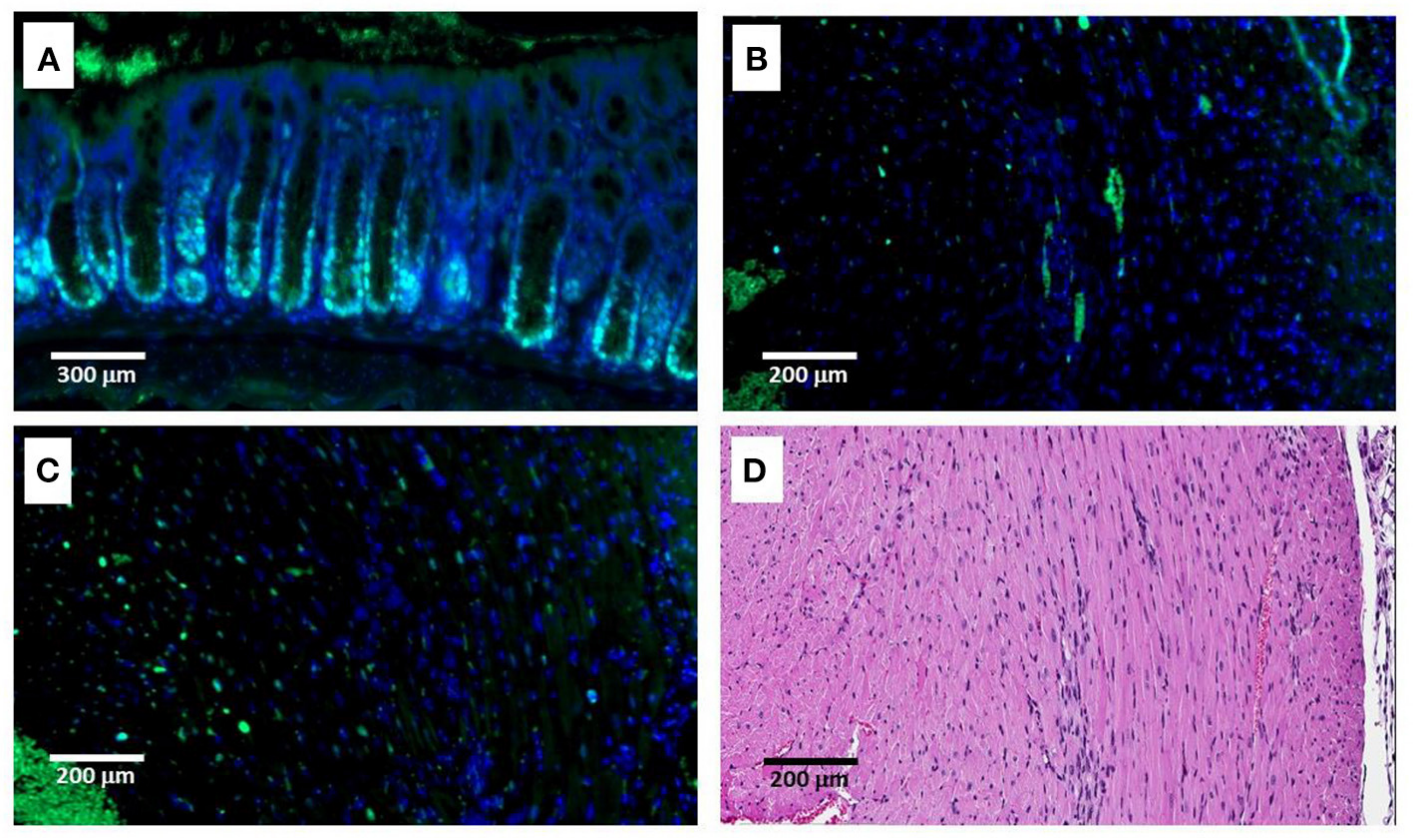
Immunofluorescence staining (IF) of proliferating cells by anti-PCNA antibody on Day 9 post-MI and intramyocardial α-gal nanoparticles injection. **(A)** Intestinal villi demonstrating multiple proliferating cells with stained nuclei at the base of the crypts. **(B,C)** IF staining of the LV by anti-PCNA antibody. Only small number of stained nuclei is observed. **(D)** H&E staining of the LV presented in **(B,C)**. Data from one mouse of 5 with similar results.

A second method for detecting proliferating cardiomyocytes was performed by anti-PCNA antibody staining of sections of treated hearts harvested on Days 9 and 11. This antibody binds to the proliferating cell nuclear antigen (PCNA) which is a DNA clamp found in nuclei at the stage of DNA synthesis. As shown in Figure 7A, nuclei of proliferating cells are readily stained by anti-PCNA at the base (crypts) of intestinal villi. In contrast, no distinct staining of cardiomyocyte nuclei was observed in the hearts treated with α-gal nanoparticles and harvested 9 or 11 days post-MI (Figures 7B,C). However, in view of the staining of a small number of nuclei observed in treated hearts both by BrdU uptake (Figures 6B,C) and by staining for PCNA (Figures 7B,C), we cannot regard these observations as conclusive evidence that there is a complete absence of proliferating cells in post-MI hearts treated with α-gal nanoparticles.

## Discussion

The present study demonstrates near-complete myocardial repair of the infarcted territory in anti-Gal producing adult GT-KO mice subjected to 30 min of LAD occlusion, followed by intramyocardial injection of α-gal nanoparticles shortly after reperfusion. In marked contrast, saline-injected controls demonstrate much larger infarcts, extensive myocardial thinning, fibrosis and scar formation in the infarct territory with corresponding impairment of LV function and persistent LV dilation. Natural regeneration of injured myocardium is observed in adult zebrafish (9) and amphibians (10, 11), whereas in mice and pigs it is observed in neonates only for a day or two after birth (12–15). The temporary post-natal natural regeneration in these mammals has led to the assumption that the mammalian heart retains a regenerative capacity, but the molecular switches are shut off in neonates shortly after birth (53, 54). Thus, it has been suggested that restoring the myocardial regenerative response in post-MI adult mammals would require “turning back the cardiac regenerative clock” (54). Presently, the mechanism of myocardium repair in post-MI adult mice treated with α-gal nanoparticles, is far from being understood. However, the similarities between natural regeneration in fish, amphibians and neonatal mice and the repair following the α-gal nanoparticles treatment in adult mice may provide some suggestions regarding this mechanism.

Activation of the complement system is common to the natural regeneration and repair induced by α-gal nanoparticles. Natural regeneration studies reported that activation of the complement cascade and upregulation of complement receptors in cells at the injury site are phenomena observed in adult zebrafish, amphibians and neonatal mice with various injuries, including heart injuries (22). Accordingly, activation of complement C5a receptor1 (C5aR1) and of C3aR1 mediates an evolutionarily conserved response that promotes cardiomyocyte proliferation after cardiac injury in all these vertebrates displaying natural regeneration (23). Moreover, regeneration of injured hearts was reduced in neonatal mice with blocking of C5a binding to its receptor C5aR1 or following genetic deletion of C5aR1 (23). It is not clear as yet what is the signal that activates the complement cascade in the course of natural regeneration, and which is absent in adult mice. It is probable, however, that activation of the complement cascade in neonates is not the result of any antigen/antibody interaction because the injury (e.g., clipping the apex of the heart) does not introduce new foreign antigens. Nevertheless, it is well-established that in anti-Gal producing adult mice treated with α-gal nanoparticles, there is an extensive activation of the complement cascade following anti-Gal binding to α-gal nanoparticles (25). The complement cleavage peptides produced by complement activation due to this antigen/antibody interaction, may trigger mechanisms for myocardium regeneration, similar to those mediating natural regeneration in neonatal mice.

A second characteristic common to natural injured heart regeneration and to post-MI myocardial repair following α-gal nanoparticles treatment is the extensive recruitment of macrophages into the injured myocardium (16–20). Nevertheless, extensive macrophages infiltration is also observed in untreated (control) post-MI hearts in adult mice (4–6, 51, 55, 56). Both mouse macrophages infiltrating into untreated post-MI hearts (51, 55) and macrophages recruited by α-gal nanoparticles in sponge disc implants (29) were found to display M2 polarization. In view of these observations, the polarization state of the infiltrating macrophages in post-MI hearts treated with by α-gal nanoparticles was not determined. Finding of M2 polarization in the latter hearts will not help in differentiating between macrophages mediating repair in the present study and those which mediated fibrosis and scar formation in untreated or saline treated hearts. In addition, there are increasing concerns that M1/M2 staining in post-MI heart histopathology may not be useful in characterization of macrophages in post-MI heart in mice (57).

Our studies have indicated however, that there are two distinct differences between these two macrophage populations: 1. Macrophages infiltrating the treated myocardium are recruited by chemotactic complement cleavage peptides such as C5a and C3a generated by anti-Gal interaction with the α-gal nanoparticles (Stage 1 in Figure 1). If the complement system is inactivated, no such recruitment is observed in skin injected with α-gal nanoparticles (25). Accordingly, macrophage recruitment is also observed in uninjured myocardium injected with α-gal nanoparticles (Figure 2B). Peak macrophage infiltration in injured hearts injected with α-gal nanoparticles is observed at Day 7 (Figure 5B). In contrast, in control saline injected post-MI myocardium, peak infiltration of macrophages is at Day 4 and the recruitment of these macrophages is mediated by substances released from ischemic myocardium such as Monocyte Chemoattractant Protein 1 (MCP-1) (58, 59) (Figure 5A). 2. Macrophages infiltrating the treated myocardium bind anti-Gal coated α-gal nanoparticles *via* Fc/Fc receptor interaction (Stage 2 in Figure 1), as shown in Figures 2C,D, whereas these nanoparticles are absent in the control post-MI myocardium. It is well-established that this Fc/Fc receptor interaction results in activation of multiple genes in macrophages, including a number of genes encoding a wide variety of cytokines (60, 61). Accordingly, interaction of recruited macrophages with anti-Gal coated α-gal nanoparticles was found to produce cytokines such as VEGF, IL1α, FGF, PDGF and CSF (25, 27). Thus, it is probable that macrophages interacting with α-gal nanoparticles within the post-MI injured myocardium also produce cytokines that mediate repair of the treated myocardium. Such cytokines may be absent in the control post-MI injured myocardium, ultimately resulting in the default fibrosis rather than restoration of the normal structure of the myocardium.

One of the unsolved questions emerging from the present study is the nature of the extensive cellular repair with cardiomyocytes in the debrided regions, occurring between Days 7 and 14 in treated post-MI hearts (Figures 5B,C). Histological examination suggests an intensive repopulation of the necrotic myocardium with healthy cardiomyocytes without evidence of cardiomyocyte hypertrophy (Figure 3F). Cardiomyocyte proliferation under the effect of cytokines produced by the pro-reparative recruited macrophages and as a result of complement cleavage peptides binding to their corresponding receptors on cardiomyocytes, is a possible explanation. However, studies on cell proliferation, using BrdU and anti-PCNA (Figures 6, 7) did not provide conclusive proof for this mechanism. This is since much less labeling of proliferating cardiomyocytes than that expected for the repopulation of the debrided regions (gray areas in Figures 5B,G) was observed by both methods.

Another possibility is that the myocardial repair is associated with recruitment and activation of cardiac-resident stem/progenitor cells, or of external mesenchymal stem cells. Stem cells may differentiate into cardiomyocytes in response to “cues” provided by the myocardial extracellular matrix (ECM) and microenvironment. Alternatively, such cells may have a paracrine effect on cell proliferation within the injured myocardium (62). Previous studies have suggested the existence of such external stem cells (e.g., bone marrow or adipose tissue stem cells) based on their ability to differentiate *in vitro* into cardiomyocytes when incubated with cardiac ECM and cardiac extracts (62–64). In addition, sponge implants in mice, which had α-gal nanoparticles, were found to contain both macrophages and colony forming cells characteristic to stem cells, within several days post-implantation (65).

In conclusion, this study is the first to demonstrate a profound reduction in myocardial infarct size, restoration of normal LV function and prevention of extensive fibrosis by intramyocardial injection of α-gal nanoparticles in adult mice after reperfusion, post-MI. The mechanism(s) mediating this repair await elucidation. Future studies include a confirmatory large animal model trial. If these dramatic benefits of α-gal nanoparticles treatment are reproduced in a large animal model of infarction and reperfusion, a clinical trial in patients with ST-elevation myocardial infarction is warranted. Following reperfusion of the occluded coronary artery with a stent in the cardiac catheterization laboratory, there is an opportunity to inject α-gal nanoparticles into the reperfused myocardium using a percutaneous catheter technique (66–68) with the goal of reducing infarct size and scar formation, improving LV function, and ultimately reducing the progression to heart failure and premature cardiac death. The large animal model planned for such study is the GT-KO pig which lacks α-gal epitope because of disruption of the α*1,3GT* gene (*GGTA1*) (42, 43) and since it produces the natural anti-Gal antibody in titers similar to those in humans, without the need of immunization (69–71).

## Data Availability Statement

The original contributions presented in the study are included in the article/Supplementary Material, further inquiries can be directed to the corresponding author.

## Ethics Statement

The animal study was reviewed and approved by IACUC Committee Rush Medical College.

## Author Contributions

UG and GS developed the method, evaluated the histological and echocardiography data, and wrote the manuscript. UG produced the nanoparticles. ZZ performed the surgical, injection work, and assisted in echocardiography studies. JC performed the echocardiography studies and histological work. JG performed the flow cytometry studies and the treatment of mice for simulation of immune parameters as in humans. All authors contributed to the article and approved the submitted version.

## Conflict of Interest

UG is the inventor of patent #8865178 “Compositions and methods for wound healing” which includes the therapy described in this manuscript. The patent is owned by the University of Massachusetts. The remaining authors declare that the research was conducted in the absence of any commercial or financial relationships that could be construed as a potential conflict of interest.

## Publisher's Note

All claims expressed in this article are solely those of the authors and do not necessarily represent those of their affiliated organizations, or those of the publisher, the editors and the reviewers. Any product that may be evaluated in this article, or claim that may be made by its manufacturer, is not guaranteed or endorsed by the publisher.

## Abbreviations

α-gal: carbohydrate antigen with the structure Galα1-3Galα1-4GlcNAc-R
BrdU: bromodeoxy uridine
ECM: extracellular matrix
FS: fractional shortening
GT-KO: mouse or pig knockout for the α1,3galactosyltransferase gene (*GTTA1*)
LAD: left anterior descending
LV: left ventricle
MI: myocardial infarction
PCNA: proliferating cell nuclear antigen
RBC: red blood cells.
