# Supplementary material for: Near Complete Repair After Myocardial Infarction in Adult Mice by Altering the Inflammatory Response With Intramyocardial Injection of α-Gal Nanoparticles
Source: Front Cardiovasc Med. 2021 Aug 25;8:719160. doi: 10.3389/fcvm.2021.719160 (PMC8425953; doi:10.3389/fcvm.2021.719160)
Supplement: Supplementary file 1 [file Data_Sheet_1.docx]

**Title:** Near complete repair after myocardial infarction in adult mice by altering the inflammatory response with intramyocardial injection of α-gal nanoparticles (**Supplementary Section for** DOI: 10.3389/fcvm.2021.719160)

**Authors:** Uri Galili PhD^a^, Zhongkai Zhu MD^b^, Jiwang Chen PhD^b^, Josef W. Goldufsky PhD^a^, Gary L. Schaer MD FAHA, FACC^a^

**Affiliations:**

^a^Department of Medicine, Rush University Medical Center, Chicago IL

^b^Department of Medicine, University of Illinois in Chicago, Chicago IL

Short Title: α-gal nanoparticles reduce myocardial infarct size

**Category:** Original Article

**Correspondence to** Uri Galili, 910 S. Michigan Ave, #904, Chicago, IL 606052; Telephone: 312-753-5997. Email: [uri.galili@rcn.com](mailto:uri.galili@rcn.com)

**SUPPLEMENTAL FIGURE LEGENDS**

**Supplemental Figure S1.** Histological sections of mouse hearts, 28 days post-MI. Sections stained with Trichrome are presented in pairs as total view of the heart (upper) and magnification of the fibrotic area (lower). **A.** Five additional saline injected post-MI hearts as in Figure 3C. **B.** Fifteen additional α-gal nanoparticles injected post-MI hearts as in Figure 3D. Scale in each group as indicated in the first figure from the left, unless specifically indicated. Corresponding planimetry analysis is included in Figure 3B.

**Supplemental Figure S2.** Histological sections of control mouse hearts 28 days post-MI. **A.** Mouse hearts post-MI receiving two injections each of 100μg in 10μl of nanoparticles lacking α-gal epitopes, i.e., nanoparticles produced from cell membranes of GT-KO pig RBC. **B.** Mice as in Figure 3D injected with α-gal nanoparticles, however, the GT-KO mice lack the anti-Gal antibody, i.e., mice that were not immunized with pig kidney membranes (PKM). Corresponding planimetry analysis is included in Figure 3B.


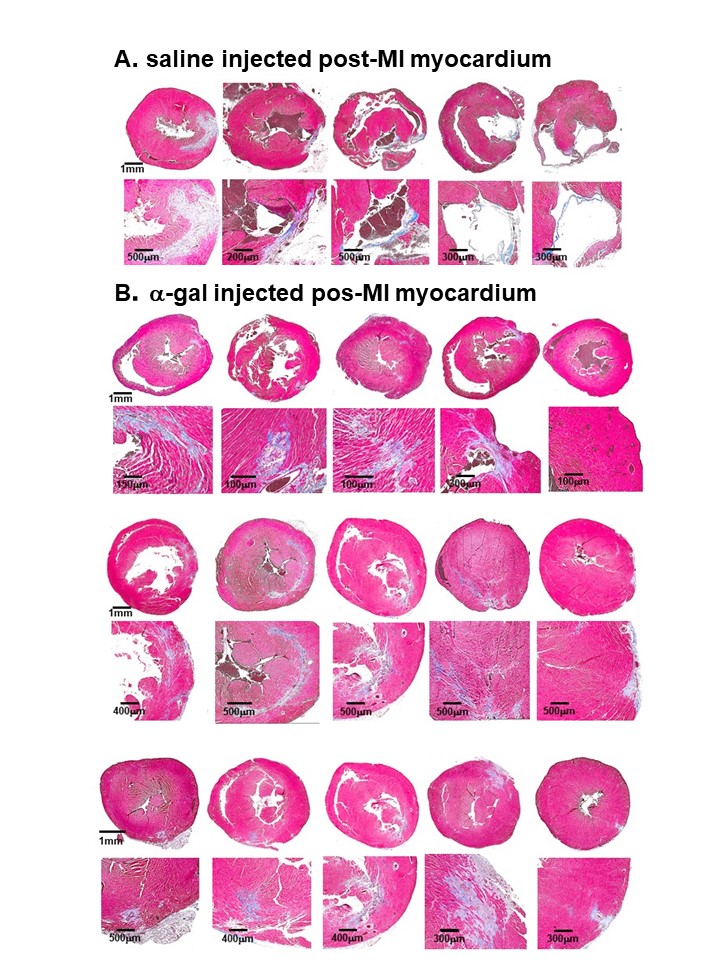


Supplemental Figure S1


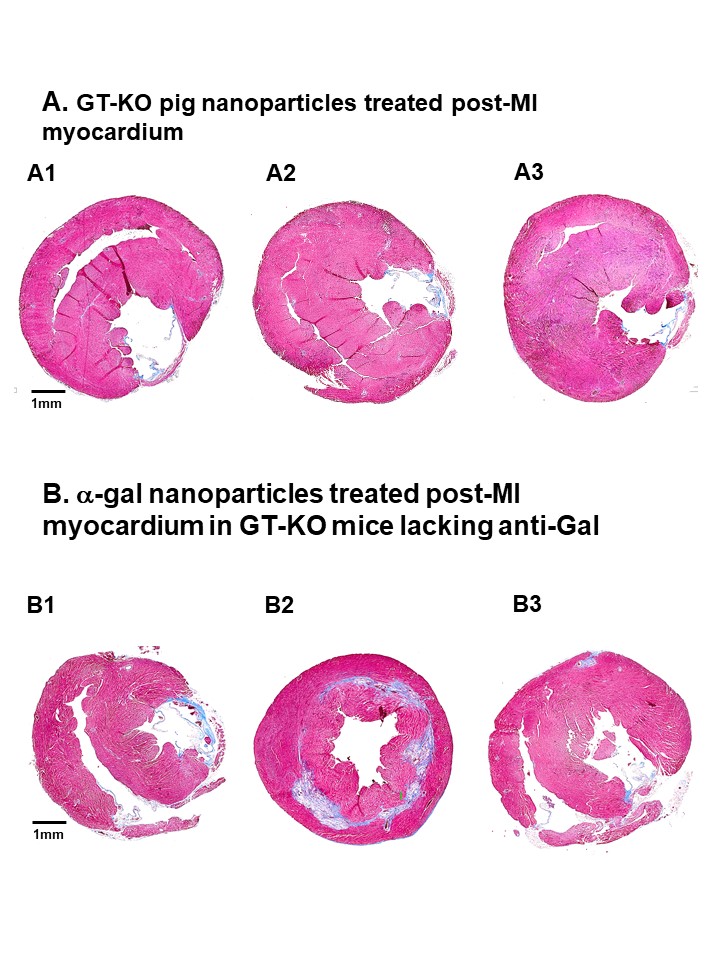


Supplemental Figure S2
